# Bidirectional genetic overlap between bipolar disorder and intelligence

**DOI:** 10.1186/s12916-022-02668-8

**Published:** 2022-11-30

**Authors:** Meng-Yuan Shang, Yong Wu, Chu-Yi Zhang, Hao-Xiang Qi, Qing Zhang, Jin-Hua Huo, Lu Wang, Chuang Wang, Ming Li

**Affiliations:** 1 Zhejiang Key Laboratory of Pathophysiology, School of Medicine, Ningbo University, Ningbo, Zhejiang, China; 2 School of Basic Medical Science, School of Medicine, Ningbo University, Ningbo, Zhejiang, China; 3 Research Center for Mental Health and Neuroscience, Wuhan Mental Health Center, Wuhan, Hubei China; 4 Key Laboratory of Animal Models and Human Disease Mechanisms of Yunnan Province, Kunming Institute of Zoology, Chinese Academy of Sciences, Kunming, Yunnan China

**Keywords:** Bipolar disorder, Intelligence, Genome-wide association study, Polygenic overlap, Shared loci

## Abstract

**Background:**

Bipolar disorder (BD) is a highly heritable psychiatric illness exhibiting substantial correlation with intelligence.

**Methods:**

To investigate the shared genetic signatures between BD and intelligence, we utilized the summary statistics from genome-wide association studies (GWAS) to conduct the bivariate causal mixture model (MiXeR) and conjunctional false discovery rate (conjFDR) analyses. Subsequent expression quantitative trait loci (eQTL) mapping in human brain and enrichment analyses were also performed.

**Results:**

Analysis with MiXeR suggested that approximately 10.3K variants could influence intelligence, among which 7.6K variants were correlated with the risk of BD (Dice: 0.80), and 47% of these variants predicted BD risk and intelligence in consistent allelic directions. The conjFDR analysis identified 37 distinct genomic loci that were jointly associated with BD and intelligence with a conjFDR < 0.01, and 16 loci (43%) had the same directions of allelic effects in both phenotypes. Brain eQTL analyses found that genes affected by the “concordant loci” were distinct from those modulated by the “discordant loci”. Enrichment analyses suggested that genes related to the “concordant loci” were significantly enriched in pathways/phenotypes related with synapses and sleep quality, whereas genes associated with the “discordant loci” were enriched in pathways related to cell adhesion, calcium ion binding, and abnormal emotional phenotypes.

**Conclusions:**

We confirmed the polygenic overlap with mixed directions of allelic effects between BD and intelligence and identified multiple genomic loci and risk genes. This study provides hints for the mesoscopic phenotypes of BD and relevant biological mechanisms, promoting the knowledge of the genetic and phenotypic heterogeneity of BD. The essential value of leveraging intelligence in BD investigations is also highlighted.

**Supplementary Information:**

The online version contains supplementary material available at 10.1186/s12916-022-02668-8.

## Background

Bipolar disorder (BD) is a highly heritable psychiatric disorder characterized by mood swings between mania/hypomania and depression [1, 2]. Early twin studies have indicated substantial contribution of a genetic component in the etiology of BD [3–5], and genetic analyses including genome-wide association studies (GWASs) have reported multiple genomic loci showing evidence of associations with risk of BD [6, 7]. To date, BD risk loci have been found to contain genes encoding ion channels, neurotransmitter transporters, and synaptic proteins [8], yet the mesoscopic phenotypes linking these pathways and BD remain largely obscure. Accumulating evidence have shown that healthy individuals or siblings carrying BD genetic risk alleles exhibited alterations in particular intermediate phenotypes (e.g., amygdala activity and cognitive function) [9–12], and studies of these intermediate phenotypes are hence believed to provide clues for the biological underpinnings of BD.

To date, growing evidence has shown a putative correlation between risk of BD and intelligence, for example, previous studies found that children with high intelligence quotient (IQ) scores had a higher chance of being diagnosed with BD in adulthood [13–15]. Further studies found that individuals with either extremely high or low school grades were more likely to be diagnosed with BD later in life compared to their peers with average performance [16], suggesting that the correlation between BD and IQ is not linear. In addition, a large-scale prospective epidemiological study of more than one million Swedish men found a “reversed–J” shaped association between intelligence and hospitalization for BD (the average follow-up period was 22.6 years, and the patients had no psychiatric comorbidities) [17]. Specifically, they found that the risk of hospitalization with any form of BD decreased as the intelligence increased; in the meantime, subjects with either the lowest IQ scores or the highest IQ scores (especially those who performed better in verbal or technical tests) had greater risk of hospitalization with pure BD [17].

These findings suggested significant associations between BD and intelligence with complicated correlation patterns. Since both BD risk and intelligence are proven to be heritable, it is plausible to hypothesize that there is a shared genetic basis between BD and intelligence. Although previous genetic correlation estimates using the Linkage Disequilibrium Score Regression (LDSC) method based on GWAS results yielded non-significant results between BD and intelligence [8], it should be noticed that the LDSC method can only capture significant correlations when multiple variants showed consistent allelic effect directions (the same or the opposite, but not mixed) in both phenotypes. Since variants associated with both BD and intelligence may have mixed directions of allelic effects between phenotypes, the putative shared genetic foundation between them is still warranted. Indeed, mixed directions of allelic effects between phenotypes with overlapped genetic basis are commonly seen [18–20], and Frei et al. described a novel statistical method, MiXeR, for precisely estimating the overall shared polygenic architecture regardless of allelic effect directions [21]. In addition, the conjunctional false discovery rate (conjFDR) analyses, which are built on an empirical Bayesian statistical framework and leverages the combined power of both GWASs, are believed to increase the opportunity of discovering novel risk loci based on GWAS summary statistics [22–27].

Using these approaches, previous studies have demonstrated extensive genetic overlap between BD and intelligence with mixed directions of allelic effects [25, 28], which seems in line with the epidemiological observations. In the present study, we repeated the MiXeR and conjFDR analyses using summary statistics from the latest GWASs of BD and intelligence, identifying both known and novel risk genes showing concordant or discordant effects between the two traits. Follow-up functional annotations and gene-set enrichment analyses found distinct molecular pathways and human phenotypes associated with “concordant loci” and “discordant loci,” respectively. These results identified genetic mechanisms explaining the phenotypic heterogeneity across the bipolar spectrum disorders and provides hints for the mesoscopic phenotypes of BD by leveraging intelligence.

## Methods

### GWAS samples

GWAS summary datasets of BD (*n* = 41,917 cases and 371,549 controls) [8] and intelligence (*n* = 269,867 individuals) [29] were retrieved from published studies. As described in the original study, the BD GWAS sample included 41,917 cases from 57 cohorts collected in Europe, North America, and Australia, and 371,549 controls from European countries [8]. The intelligence GWAS data included 14 datasets from Europe and North America, and a common latent *g* factor underlying multiple dimensions of cognitive functioning was calculated and applied to operationalize the cohorts with intelligence measured using distinct approaches [29]. The information about the effect allele, effect size (beta or odds ratio), standard error, and *P* value were obtained from the GWAS studies.

### Statistical analyses

The genome-wide genetic correlation (*r*_g_) of BD with intelligence was calculated using LDSC [30]. MiXeR (version v1.3) was used to construct a bivariate causal mixture model to estimate the total number of shared and trait-specific causal variants between BD and intelligence based on GWAS summary statistics [21, 31]. MiXeR results are presented as a Venn diagram of the shared and unique polygenic components across BD and intelligence, and the dice coefficient score (polygenic overlap measure in the 0~100% scale) were also computed [18].

The conditional quantile-quantile (Q-Q) plots were generated to provide a visual pattern of enrichment in single nucleotide polymorphism (SNP) associations between BD and intelligence. As described in the previous study [32], the Q-Q plots computed the empirical cumulative distributions of *P* values in the primary phenotype for all SNPs and for subsets of SNPs (e.g., *P* ≤ 0.1, *P* ≤ 0.01, *P* ≤ 0.001, respectively) in the secondary phenotype. Increased degree of leftward deflection, from the expected null line as the association significance increases in a phenotype, suggests enrichment of associations of the other phenotype [32].

We next conducted the conjFDR analyses to characterize the genomic loci and SNPs jointly associated with both BD and intelligence. As described in the previous studies [32, 33], this method re-adjusted the GWAS summary statistics in the primary phenotype (e.g., BD) by leveraging pleiotropic enrichment with the GWAS summary statistics in the secondary phenotype (e.g., intelligence). The conditional FDR (condFDR) estimates were calculated for each variant in the primary phenotype using the stratified empirical cumulative distribution function. This process was then performed again with the primary and secondary phenotypes switched, and conjFDR was defined as the maximum of the two condFDR values [32, 33]. During the conjFDR analyses, all *P* values in the original GWAS datasets were corrected for genomic inflation, since the empirical null distributions of SNP associations in GWASs might be affected by population stratification [32]. Random pruning of SNPs was performed throughout the 500 iterations in both the conditional Q-Q plots and conjFDR analyses to minimize inflation resulted from linkage disequilibrium (LD) dependency, and one randomly-returned representative SNP for each LD-independent block was retained after every pruning iteration (cluster of SNPs with *r*^2^ > 0.1). As recommended in the previous study [18], we remained only one signal in the highly extended major histocompatibility complex (MHC) region (hg19, chr6:26M-34M) to minimize the impacts of complicated LD patterns in this genomic area. SNPs with a conjFDR < 0.01 were considered statistically significant.

### Functional annotations of the genomic risk loci

To identify genes associated with the risk loci, we conducted expression quantitative trait loci (eQTL) analyses of all the significant SNPs in each independent genomic region (conjFDR < 0.01 and at *r*^2^ < 0.2), to identify genes associated with the risk loci in multiple datasets. We used datasets of postmortem postnatal human brain tissues (dorsolateral prefrontal cortex (DLPFC) or hippocampus) such as psychENCODE (*n* = 1387 for DLPFC) [34], BrainMeta (*n* = 2865 for cortex) [35], BrainSeq (*n* = 477 for hippocampus) [36] and Genotype-Tissue Expression (GTEx; *n* = 175 for DLPFC and *n* = 165 for hippocampus) [37], datasets of prenatal cortex tissues from O’Brien et al. (*n* = 120) [38] and Walker et al. (*n* = 201) [39] studies, as well as single-cell eQTLs resources (including mature midbrain dopaminergic neurons (from 175 donors) and serotonergic-like neurons (from 161 donors) differentiated from human induced pluripotent stem cells (iPSC) lines [40]; and excitatory neurons and inhibitory neurons from 196 individuals by single nuclei RNA-sequencing [41]). Detailed information about the sample characteristics, expression quantification, and normalization, as well as statistical analysis in each eQTL dataset, can be found in the original publications. The psychENCODE and BrainSeq datasets only provided SNP associations passing genome-wide level of statistical significance (false discovery rate (FDR) < 0.05 in psychENCODE and FDR < 0.01 in BrainSeq), we hence directly retrieved the significant eQTL associations. For the other eQTL datasets, we empirically considered the genes with eQTL *P* < 0.001 to be significant. We did not perform eQTL analysis in the MHC region (hg19, chr6:26M-34M) given its complicated LD patterns and potential mapping problems using short-reads sequencing.

### Enrichment analyses of the risk genes

For the risk genes identified by eQTL analyses, we conducted enrichment analyses of Gene Ontology (GO), Reactome pathway [42], UniProt keywords [43], and Monarch Human Phenotype Ontology (HPO) [44] using the STRING dataset (version 11.5) [45].

## Results

### Polygenic overlap between BD and intelligence

**Fig. 1:**
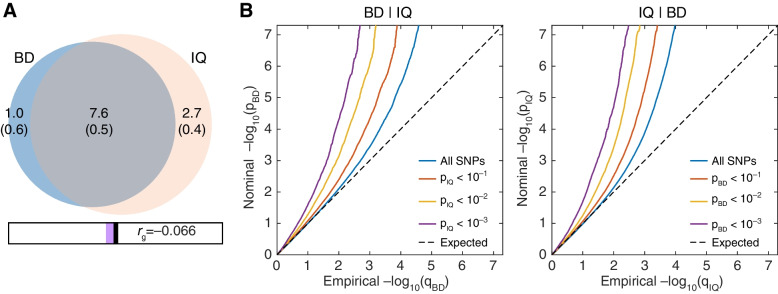
Polygenic overlap between bipolar disorder (BD) and intelligence (IQ). **A** The estimated number of causal variants shared (grey) between BD and IQ calculated by MiXeR, and the genetic correlation (*r*_g_= −0.066) is estimated by LDSC. **B** Conditional Q-Q plots of nominal versus empirical −log_10_ transformed *P* values in the primary phenotype as a function of significance of SNP associations with the secondary phenotype at the level of *P* ≤ 1.00 (all SNPs, blue lines), *P* ≤ 0.1 (red lines), *P* ≤ 0.01 (yellow lines) and *P* ≤ 0.001 (purple lines). The dashed line is the expected Q-Q plot under the null hypothesis

The polygenic overlap (i.e., shared causal variants) between BD and intelligence was assessed using MiXeR based on GWAS summary statistics. The bivariate MiXeR analysis revealed that approximately 8.6K (standard deviation (SD) = 0.2K) variants influenced BD, and approximately 10.3K (SD = 0.3K) variants influenced intelligence. Intriguingly, 7.6K (SD = 0.5K) variants influenced both BD and intelligence, and the overall measure of polygenic overlap between BD and intelligence was 80.3% (standard error (SE) = 4.9%) on a 0–100% scale (quantified as the Dice coefficient) (Fig. 1A).

We then generated conditional quantile-quantile (Q-Q) plots conditioning BD on intelligence and *vice versa*, to further describe the pleiotropic enrichment of SNP associations between BD and intelligence. The obvious leftward deflection of the curves for both BD-given intelligence and intelligence-given BD suggested strong enrichment of the significant variants for one trait in the other. Notably, SNPs with higher significance for the conditional trait exhibited greater leftward deflection in both plots, confirming the substantial polygenic overlap between BD and intelligence (Fig. 1B).

### Shared genetic loci between BD and intelligence

Both MiXeR and the Q-Q plots indicated significant polygenic overlap between BD and intelligence, but no significant genetic correlation was observed between them (LDSC *r*_g_ = −0.066, *P* = 0.062, Fig. 1A). Since LDSC analyses only reported significant genetic correlations with the premise that there were numerous SNPs showing associations with both phenotypes in concordant direction of allelic effects, we suspected that the shared variants between BD and intelligence had mixed directions of allelic effects. Indeed, MiXeR analysis showed that the “concordant variants” took up only 47% (SE = 0.4%) of the shared genetic components (7.6K variants) between BD and intelligence.

**Fig. 2:**
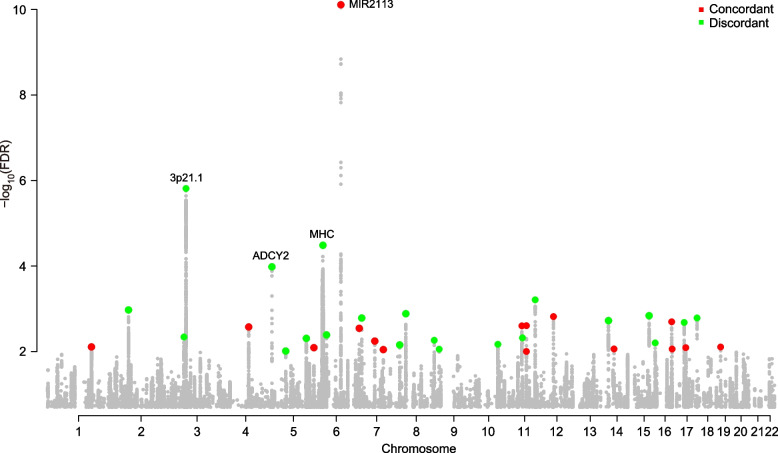
Manhattan plots for conjFDR analyses. SNPs jointly associated with BD and intelligence in the conjFDR analysis (conjFDR < 0.01). Lead SNPs in each independent risk loci with the same directions of allelic effects between BD and intelligence are marked in red, and lead SNPs in each independent risk loci with opposite directions of allelic effects between BD and intelligence are marked in green

We hence applied the genetic pleiotropy-informed conjFDR method [33], which identifies loci associated with both BD and intelligence regardless of the allelic effect directionality with boosted statistical power. We identified 37 distinct genomic loci (*r*^2^ < 0.2) that were jointly associated with BD and intelligence with a conjFDR < 0.01 (Fig. 2 and Table 1), including 24 independent loci that were not identified in the original BD GWAS [8]. Further computation of the z-scores of these loci revealed that 16 SNPs exhibited the same directions of effect between BD and intelligence (i.e., one allele predicted a higher risk of BD and greater intelligence), while 21 SNPs had the opposite directions (Fig. 2 and Table 1). This finding is consistent with a previous study describing loci jointly associated with BD and intelligence [25], albeit that study reported fewer joint risk loci.

**Table 1:** Independent genomic loci significantly associated with BD and intelligence at a conjunctional false discovery rate <0.01

	CHR	POS	SNP	conjFDR	Novel	A1	A2	OR_BD	P_BD	BETA_IQ	P_IQ	OR_SCZ	P_SCZ	eQTL	Nearby genes
**Concordant**	1	174380810	rs7548540	7.77E−03	Yes	G	A	1.040	3.17E−05	0.014	3.06E−07	0.993	0.449	Adult	RABGAP1L
	4	106201556	rs2647256	2.66E−03	Yes	T	C	1.053	6.06E−06	0.020	4.43E−09	1.033	0.00255	Adult	TET2
	5	169271149	rs12515788	8.09E−03	No	T	C	1.060	4.26E−06	0.016	1.66E−05	1.008	0.491		DOCK2
	6	98565211	rs1487445	7.78E−11	No	T	C	1.077	1.48E−15	0.031	3.27E−29	1.032	0.000229		MIR2113
	7	11907220	rs2883598	2.86E−03	No	A	G	1.065	2.08E−10	0.014	2.77E−06	1.022	0.0188		THSD7A
	7	71778628	rs11766995	5.68E−03	Yes	T	C	1.108	1.34E−05	0.034	8.92E−06	1.088	0.000208	Adult	CALN1
	7	104667334	rs9655780	9.00E−03	Yes	G	A	1.054	2.22E−05	0.016	2.00E−05	0.973	0.0193	Adult	MLL5, SRPK2
	11	61607168	rs174582	2.50E−03	No	G	A	1.059	5.46E−07	0.016	2.22E−06	1.017	0.109	Adult, fetal	FADS1, FADS2
	11	79129616	rs12273727	9.93E−03	No	G	A	1.054	1.09E−05	0.015	2.38E−05	1.029	0.00904		ODZ4
	11	79153448	rs11237837	2.48E−03	No	A	G	1.049	4.39E−06	0.015	2.19E−06	1.033	0.000893		ODZ4
	12	49478658	rs6580698	1.52E−03	Yes	C	T	1.047	2.62E−06	0.019	5.08E−12	1.011	0.204	Adult, fetal	MLL2, RHEBL1, DHH
	14	51692699	rs12431939	8.67E−03	Yes	A	G	1.057	1.04E–05	0.016	1.87E−05	1.029	0.0136	Adult	TRIM9, TMX1
	16	70756066	rs7196032	2.01E−03	Yes	T	C	1.046	3.98E−06	0.015	4.47E−08	1.000	0.967	Adult	MTSS1L, VAC14
	16	72450482	rs1013982	8.71E−03	Yes	G	A	1.042	3.79E−05	0.013	5.16E−06	1.009	0.347		AC004158.2
	17	34892731	rs11650008	8.09E−03	Yes	C	A	1.040	3.38E−05	0.016	3.62E−09	1.040	7.08E−06	Adult	MYO19, PIGW, GGNBP2
	19	13116172	rs17706798	7.81E−03	Yes	C	T	1.041	3.19E−05	0.013	1.08E−05	1.037	6.62E−05		DAND5, NFIX
**Discordant**	2	73552542	rs7573275	1.06E−03	Yes	A	G	1.051	1.55E−06	−0.017	1.24E−08	1.041	2.44E−05	Adult, fetal	U6, ALMS1
	3	44817181	rs7611773	4.55E−03	Yes	C	T	1.043	6.76E−06	−0.012	6.11E−06	1.004	0.622	Adult, Fetal	ZNF502, ZNF501, KIF15
	3	52618319	rs12487445	1.54E−06	No	A	C	1.062	2.44E−10	−0.018	3.31E−11	1.062	7.69E−12	Adult, fetal	3p21.1 region
	5	7519298	rs17826816	1.04E−04	No	G	A	1.065	1.32E−08	−0.018	1.68E−08	1.018	0.0735		ADCY2
	5	60727990	rs10939902	9.72E−03	Yes	C	T	1.039	4.50E−05	−0.013	4.22E−06	1.072	7.36E−16	Adult	ZSWIM6
	5	140142174	rs2531337	4.91E−03	Yes	A	C	1.048	1.56E−05	−0.016	7.97E−07	1.048	3.30E−06	Adult, fetal	PCDHA1
	6	29342775	rs3749971	3.27E−05	No	G	A	1.106	8.91E−10	−0.030	3.06E−09	1.191	2.10E−28		MHC region
	6	43203583	rs74371173	4.08E−03	Yes	T	C	1.079	6.17E−06	−0.024	5.06E−06	1.065	5.33E−05	Adult, fetal	SRF, CUL9, TTBK1
	7	21483605	rs2237303	1.64E−03	No	G	A	1.054	6.26E−08	−0.014	1.12E−06	1.029	0.00129	Adult	SP4
	8	10266706	rs12550717	6.98E−03	No	A	G	1.046	4.60E−06	−0.013	1.28E−05	1.038	2.60E−05	Adult	MSRA
	8	33863561	rs79445414	1.30E−03	No	C	T	1.121	8.07E−07	−0.035	7.71E−07	1.131	2.80E−08		RP11-317N12.1
	8	143347922	rs4601464	5.44E−03	Yes	C	T	1.041	1.82E−05	−0.013	1.41E−06	1.064	1.18E−12	Adult	TSNARE1
	9	16730258	rs59234174	8.78E−03	Yes	T	C	1.056	2.46E−05	−0.016	1.91E−05	1.034	0.00520		BNC2
	10	101997490	rs11190431	6.76E−03	Yes	A	G	1.083	2.55E−05	−0.024	6.91E−07	1.046	0.00811	Adult	ERLIN1, CHUK, BLOC1S2
	11	63941110	rs11608180	4.77E−03	No	G	A	1.102	6.17E−08	−0.025	6.62E−06	1.047	0.00541	Adult	FERMT3, TRPT1, FKBP2
	11	112957783	rs56331084	6.15E−04	Yes	A	C	1.060	6.99E−07	−0.018	1.78E−07	1.050	7.78E−06	Excitatory	NCAM1
	14	30208630	rs1959440	1.89E−03	Yes	T	G	1.049	3.51E–07	−0.013	1.41E−06	1.055	1.26E−09	Adult	PRKD1
	15	82451554	rs62011975	1.45E−03	Yes	C	T	1.056	2.44E−06	−0.018	4.55E−08	1.044	3.33E−05	Adult, fetal	EFTUD1
	16	7225505	rs4630565	6.27E−03	Yes	T	C	1.045	4.12E−06	−0.012	1.06E−05	1.029	0.00130		RBFOX1
	17	28864382	rs216472	2.09E−03	Yes	G	A	1.044	4.22E−06	−0.014	1.74E−07	1.032	0.000227	Adult, fetal	CPD, GOSR1
	17	78485226	rs7207843	1.64E−03	Yes	C	T	1.057	1.65E−06	−0.017	1.11E−06	1.052	2.30E−06	Adult, fetal	NPTX1, RPTOR

**Fig. 3:**
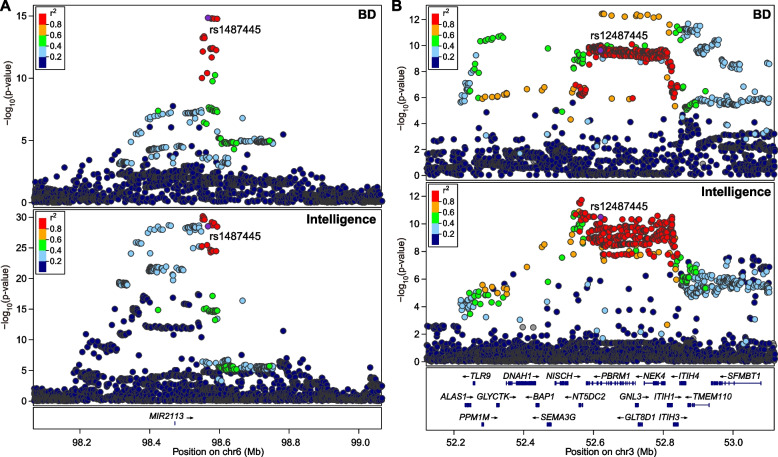
Associations of SNPs at MIR2113 on chromosome 6 (**A**) and 3p21.1 region (**B**) with risk of BD and intelligence in the GWAS datasets. A physical map of the region is given and depicts known genes within the region, and the LD is defined based on the SNP rs1487445 (**A**) and rs12487445 (**B**), respectively

We then performed a detailed genomic mapping analysis of the 37 shared loci between BD and intelligence. The locus with the strongest concordant effect on both the risk of BD and intelligence was in MIR2113 on chromosome 6 (rs1487445, conjFDR = 7.78×10^−11^), and the T-allele of rs1487445 predicted both increased risk of BD (odds ratio (OR) = 1.077, *P* = 1.48×10^−15^) and higher IQ scores (beta = 0.031, *P* = 3.27×10^−29^) (Fig. 3A). The variant with the strongest opposite effects on BD and intelligence is in the chromosome 3p21.1 region (rs12487445, conjFDR = 1.54×10^−6^), and the A-allele of rs12487445 predicted increased risk of BD (OR = 1.062, *P* = 2.44×10^−10^) but lower IQ scores (beta = −0.018, *P* = 3.31×10^−11^) (Fig. 3B). The locus showing the second strongest opposite effects was in the highly extended MHC region (rs3749971, conjFDR = 3.27×10^−5^), and the G-allele of rs3749971 predicted increased risk of BD (OR = 1.106, *P* = 8.91×10^−10^) while lower IQ scores (beta = −0.030, *P* = 3.06×10^−9^) (Fig. S1). Another locus with the opposite directions of allelic effects between BD and intelligence was in the gene ADCY2 (rs17826816, conjFDR = 1.04×10^−4^), and its G-allele was linked to higher risk of BD (OR = 1.065, *P* = 1.32×10^−8^) but lower IQ scores (beta = −0.018, *P* = 1.68×10^−8^) (Fig. S2).

Since BD and schizophrenia exhibit strong genetic correlations [46], and schizophrenia also has substantial polygenic overlap with intelligence [25], there is the possibility that the identified BD risk loci through leveraging intelligence are not exclusively associated with BD. We verified this by examining the aforementioned 37 BD risk SNPs in the largest schizophrenia GWAS in Europeans (74,776 cases and 101,023 controls) [47], we found that 15 of these loci were also associated with schizophrenia in the same allelic directions (Table 1). Notably, 13 of the 21 SNPs showing opposite directions of effect between BD and intelligence also exhibited significant associations with schizophrenia; by contrast, only 2 of the 16 SNPs showing the same directions of effect between BD and intelligence were associated with schizophrenia. Therefore, although both BD and schizophrenia have polygenic overlap with intelligence, the potential genetic mechanisms regulating intelligence in these two disorders are likely divergent.

### Risk genes and functional annotations in the significant genomic loci

Since majority of the risk SNPs for BD are in the noncoding genomic regions, functional annotations of them are urgently needed. Accumulating evidence suggests that noncoding SNPs affect gene transcription and splicing likely in a cell type- and developmental stage-specific manner [7, 48]. We thus examined the regulatory effects of the BD risk SNPs using datasets containing human eQTL results in postnatal DLPFC and hippocampal tissues, prenatal cortex tissues, as well as different types of brain cells. In summary, 25 of the 37 risk loci had eQTL associations in at least one dataset (Table 1); 9 of the 16 risk loci showing concordant effects on BD and intelligence contained eQTL-associated risk genes, while 16 of the 21 loci showing opposite effects on BD and intelligence contained eQTL associated risk genes. There were no overlapped eQTL risk genes between the “concordant loci” and “discordant loci”.

**Fig. 4:**
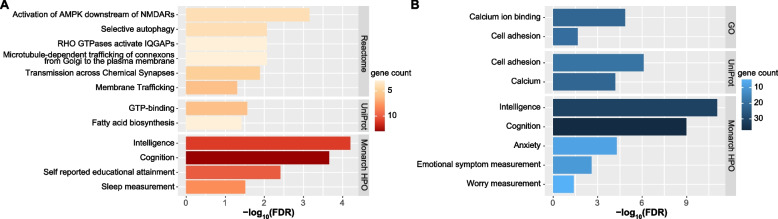
Enrichment analyses of the eQTL genes in the “concordant loci” (**A**) and “discordant loci” (**B**). The enrichment analyses were conducted in the STRING website, and default parameters were applied

Specifically, 37 protein-coding genes were significantly affected by SNPs in the loci showing concordant effects on BD and intelligence (Table S1). Although GO analysis of these genes did not reveal any significant enrichment, Reactome pathway analysis found that their proteins were significantly enriched in the pathways of “Activation of AMPK downstream of NMDARs,” “Microtubule-dependent trafficking of connexons from Golgi to the plasma membrane,” “RHO GTPases activate IQGAPs,” “Selective autophagy,” “Transmission across Chemical Synapses,” and “Membrane Trafficking” (Fig. 4A and Table S2). UniProt keywords analysis suggested that these proteins were significantly enriched in the terms of “GTP-binding” and “Fatty acid biosynthesis” (Fig. 4A and Table S2). Monarch HPO analysis found that these genes were enriched in the terms “Intelligence,” “Cognition,” and “Self reported educational attainment.” Intriguingly, we found that these genes also showed significant enrichment in the term “Sleep measurement” (Fig. 4A and Table S2).

We identified 135 protein-coding genes affected by SNPs in the genomic loci showing opposite effects on BD and intelligence (Table S3). GO analysis of these genes revealed significant enrichment in the terms “Cell adhesion” and “Calcium ion binding”, whereas Reactome pathway analysis did not identify any significantly enriched pathways (Fig. 4B and Table S4). Similarly, UniProt keywords analysis found significant enrichment of these proteins in the terms “Cell adhesion” and “Calcium” (Fig. 4B and Table S4). Monarch HPO analysis found that these genes were enriched in the terms “Intelligence” and “Cognition.” It should be noticed that these genes were also significantly enriched in “Anxiety,” “Emotional symptom measurement,” and “Worry measurement” (Fig. 4B and Table S4).

## Discussion

A previous study by Gale et al. [17] found elevated risk of developing BD in subjects with either lower or higher IQ scores, and extensive genetic overlap between BD and intelligence with mixed directions of allelic effects was demonstrated [25, 28]. In the present study, we have identified multiple genomic loci and risk genes correlated with both BD and intelligence, with either the same or opposite directions of allelic effects. These results provide possible explanations for the higher prevalence of BD in subjects with either low or high intelligence, and highlight the etiological heterogeneity of BD.

By leveraging pleiotropic enrichment between BD and intelligence using the conjFDR method, we herein identified 37 loci jointly associated with BD and intelligence (16 loci showed the same allelic effect directions, and 21 loci showed opposite directions) based on large-scale GWAS summary statistics, and 24 loci were not identified in the original BD GWAS [8]. We noticed that one of the most significant loci, which showed the same directions of allelic effects between BD and intelligence, was in MIR2113 on chromosome 6 (rs1487445, conjFDR = 7.78×10^−11^). Notably, a recent study found that a variant rs77910749, which is in complete LD with rs1487445 (*r*^2^ = 1.00 in Europeans) and resides within a highly conserved putative enhancer LC1 in the upstream region of POU3F2 [49], could alter LC1 enhancer activity and POU3F2 expression during neurodevelopment in embryonic mouse brain and human iPSC-derived cerebral organoids. Intriguingly, rs77910749 knock-in mice exhibited behavioral defects in sensory gating [49], which is an amygdala-dependent endophenotype commonly seen in BD patients [50].

Despite that lower intelligence and higher intelligence are both linked to increased risk of BD, we speculate that the molecular mechanisms underlying the two conditions are distinct. Indeed, functional annotations revealed that no overlap of the genes affected by the “concordant loci” and those affected by the “discordant loci.” Further analyses found that genes related to the “concordant loci” were significantly enriched in synapses related pathways, whereas genes related to the “discordant loci” were significantly enriched in the biological processes of “Cell adhesion” and “Calcium ion binding.” More intriguingly, although both sets of genes showed significant enrichment in the terms “Intelligence” and “Cognition,” the “concordant genes” also showed enrichment in “Sleep measurement,” whereas the “discordant genes” were rather enriched in “Anxiety,” “Emotional symptom measurement,” and “Worry measurement.” These results suggested that the “concordant genes” were likely related to the dysrhythmia in BD, while “discordant genes” were possibly involved in the abnormal emotional behaviors. Therefore, further investigations into genes shared by intelligence and BD, either with the same or opposite directions of allelic effects, will likely extend our knowledge of this disorder and biological mechanisms of the human brain.

Nonetheless, we acknowledged the potential limitation that the shared loci between BD and intelligence can not fully explain their nonlinear relationships in epidemiological observations, as other factors (e.g., family and social environment, humanistic culture, and education) may also affect these traits. In addition, our analyses were based on samples from European ancestry, despite the results being intriguing, validations in other ethnic populations are necessary in the future.

## Conclusions

We observed substantial polygenic overlap between BD and intelligence and identified multiple loci associated with BD and intelligence with mixed directions of allelic effects. Enrichment analyses suggested different biological processes related to the “concordant genes” and the "discordant genes", providing hints for the mesoscopic phenotypes of BD and relevant biological mechanisms. An appealing hypothesis, that whether BD patients with lower intelligence exhibit more severe emotional disturbance, while BD patients with higher intelligence have more frequent dysrhythmia, is of great interest for further clinical validations.

## Supplementary Information

**Additional file 1.**

**Additional file 2.**

## Data Availability

All the GWAS data and statistical software used in this study were publicly available (which can be accessed through the following URLs), and all the generated results in this study were provided in the main text and supplemental data. URLs: Bipolar disorder GWAS: 10.6084/m9.figshare.14102594 BrainMeta: https://yanglab.westlake.edu.cn/data/brainmeta/cis_sqtl/ BrainSeq: http://eqtl.brainseq.org/phase2/eqtl/ conjFDR: https://github.com/precimed/pleiofdr eQTLs in Bryois et al. study: https://zenodo.org/record/6104982#.Y3TUGnZBxD8 eQTLs in Jerber et al. study: https://zenodo.org/record/4333872#.Y3TUgnZBxD8 eQTLs in O’Brien et al. study: 10.6084/m9.figshare.6881825 GTEx: http://www.gtexportal.org/home/ Intelligence GWAS: https://ctg.cncr.nl/software/summary_statistics LDSC: https://github.com/bulik/ldsc LocusZoom: http://locuszoom.org MiXeR: https://github.com/precimed/mixer PsychENCODE: http://www.psychencode.org/ Schizophrenia GWAS: 10.6084/m9.figshare.19426775 STRING: https://cn.string-db.org/

## Abbreviations

BD: Bipolar disorder
condFDR: Conditional false discovery rate
conjFDR: Conjunctional false discovery rate
DLPFC: Dorsolateral prefrontal cortex
eQTL: Expression quantitative trait loci
FDR: False discovery rate
GO: Gene Ontology
GWAS: Genome-wide association studies
GTEx: Genotype-Tissue Expression
HPO: Human Phenotype Ontology
IQ: Intelligence quotient
iPSC: Induced pluripotent stem cells
LD: Linkage disequilibrium
LDSC: Linkage Disequilibrium Score Regression
MHC: Major histocompatibility complex
OR: Odds ratio
Q-Q: Quantile-quantile
SD: Standard deviation
SE: Standard error
SNP: Single nucleotide polymorphism
