# Supplementary material for: Bidirectional genetic overlap between bipolar disorder and intelligence
Source: BMC Med. 2022 Nov 30;20:464. doi: 10.1186/s12916-022-02668-8 (PMC9710050; doi:10.1186/s12916-022-02668-8)

Figure S1. Genetic associations of major histocompatibility complex (MHC) genes region with BD risk and intelligence in the GWAS datasets. A physical map of the region is given and depicts known genes within the region, and the LD is defined based on the SNP rs3749971.

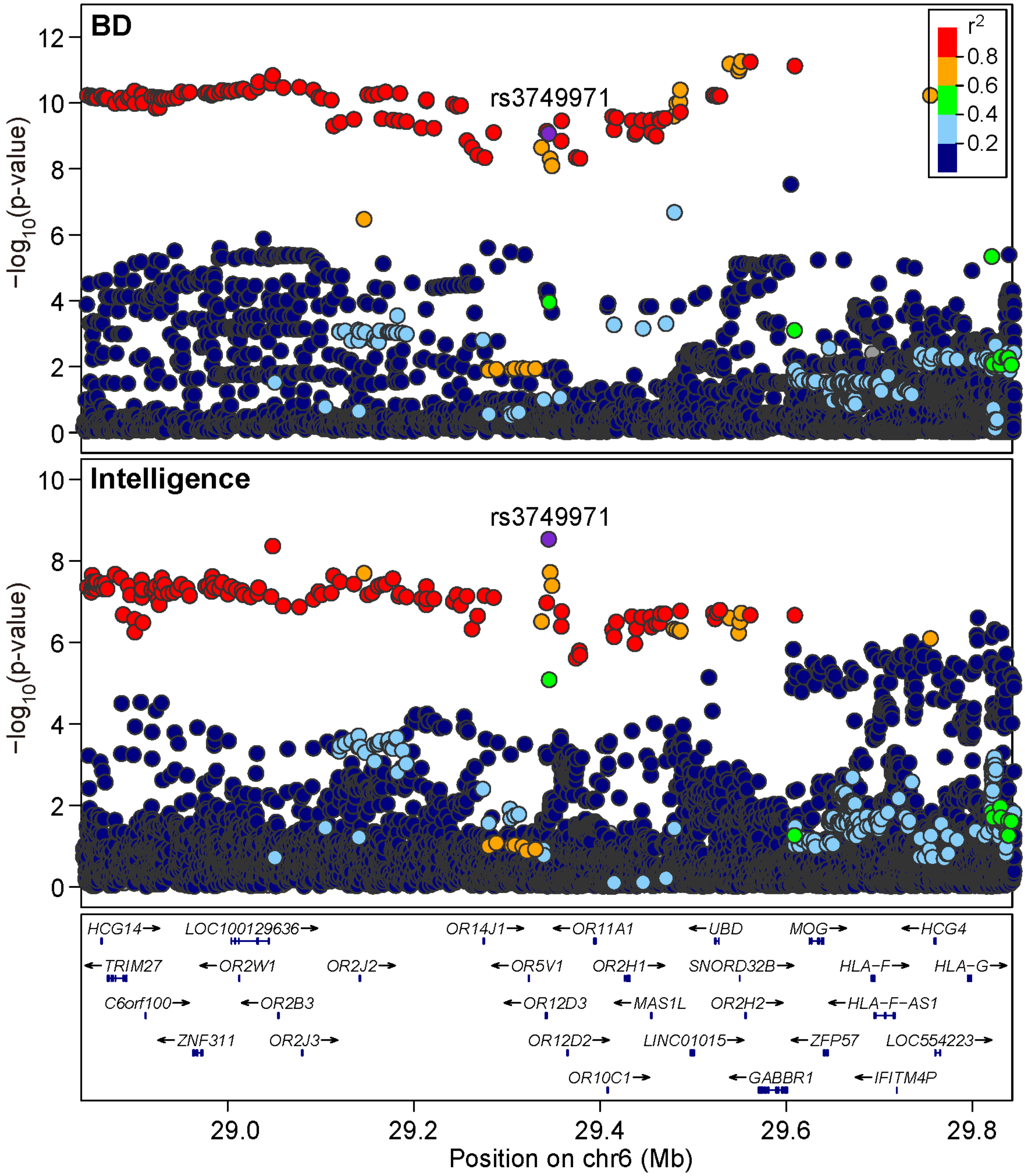

Figure S2. Genetic associations of ADCY2 region with BD risk and intelligence in the GWAS datasets. A physical map of the region is given and depicts known genes within the region, and the LD is defined based on the SNP rs17826816.

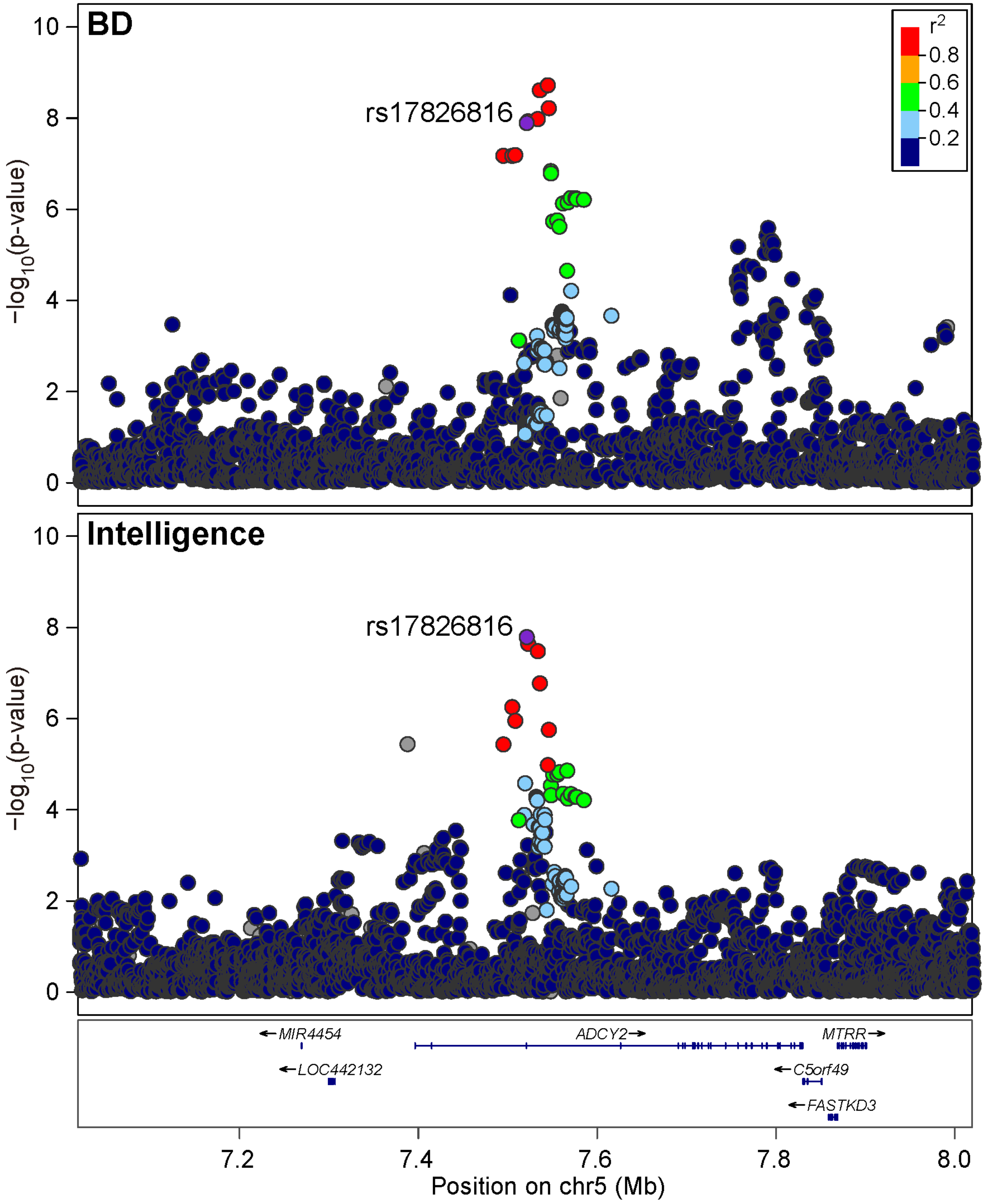

Supplement: Supplementary file 1 — Additional file 1. [file 12916_2022_2668_MOESM1_ESM.pdf]
